# Differential effects of ginsenosides on Ca^2+^ regulation in rotenone-treated neuronal and microglial cells

**DOI:** 10.1371/journal.pone.0356602

**Published:** 2026-08-18

**Authors:** Jiwoo Shin, Geun Hee Seol, Yoo Jin Kim, Hayeong Jeon

**Affiliations:** 1 Department of Basic Nursing Science, College of Nursing, Korea University, Seoul, Republic of Korea; 2 BK21 FOUR Program of Transdisciplinary Major in Learning Health Systems, Graduate School, Korea University, Seoul, Republic of Korea; University of Bergen: Universitetet i Bergen, NORWAY

## Abstract

Parkinson’s disease is one of the most common neurodegenerative disorders, and the pesticide rotenone is widely used to model Parkinson’s disease in experimental studies. Rotenone-induced mitochondrial dysfunction is associated with oxidative stress and intracellular Ca^2+^ ([Ca^2+^]ᵢ) dysregulation in neuronal systems. L-type Ca^2+^ channels (LTCCs) contribute to Ca² ⁺ influx under oxidative stress conditions, but pharmacological inhibition of LTCCs is limited by cardiovascular side effects. Phospholipase D (PLD) has also been implicated in cellular stress-associated signaling pathways. Ginsenosides are known to influence Ca² ⁺ -related signaling; however, differences among ginsenosides in neuronal and microglial Ca² ⁺ regulation have not been fully characterized. Therefore, this study aimed to investigate the effects of ginsenosides on intracellular Ca² ⁺ homeostasis and oxidative stress of rotenone-exposed SH-SY5Y neuronal cells and BV2 microglial cells. Cell viability, superoxide dismutase (SOD) activity, interleukin-6 (IL-6) levels, intracellular Ca² ⁺ influx, and malondialdehyde (MDA) levels were evaluated. Intracellular Ca² ⁺ was measured using Fura-2 AM ratiometric fluorescence analysis. The effects of ginsenosides Rg1, Rg2, and Rd were examined using pharmacological inhibitors targeting PLD, LTCCs, and protein kinase A (PKA). Rotenone reduced cell viability and SOD activity, while increasing IL-6 levels, [Ca^2+^]ᵢ influx, and MDA levels in both cell types. Treatment with ginsenosides immediately before rotenone exposure attenuated rotenone-induced increases in [Ca^2+^]ᵢ and MDA levels. Ca² ⁺ responses in both SH-SY5Y and BV2 cells were sensitive to PLD inhibition. In SH-SY5Y cells, responses were additionally sensitive to LTCC inhibition, whereas BV2 cells showed weaker LTCC-associated pharmacological responses. Rd showed broader pharmacological sensitivity involving PLD-, LTCC-, and PKA-associated components, whereas Rg1 and Rg2 showed predominantly PLD-associated response patterns. These findings suggest that ginsenosides differentially modulate rotenone-associated Ca² ⁺ dysregulation through PLD-associated pharmacological pathways in a cell type-dependent manner. Overall, the present findings demonstrate distinct pharmacological response profiles among structurally different ginsenosides under rotenone-induced oxidative stress conditions.

## Introduction

Parkinson’s disease (PD) is a progressive neurodegenerative disorder characterized by the selective loss of dopaminergic neurons in the substantia nigra. Levodopa remains the standard pharmacotherapy to replenish dopamine deficiency; however, chronic administration is associated with complications including dyskinesia, orthostatic hypotension, and cardiac arrhythmia [1]. Increasing evidence indicates that reactive oxygen species (ROS)-associated intracellular Ca² ⁺ overload plays a pivotal role in neuronal vulnerability under parkinsonian conditions [2]. Therefore, pharmacological regulation of pathological Ca² ⁺ influx under oxidative stress conditions has attracted increasing attention as a potential therapeutic strategy in experimental PD models.

Rotenone, a mitochondrial complex I inhibitor, is widely used to induce Parkinsonian cellular stress through mitochondrial dysfunction and excessive ROS generation [3]. Previous studies have shown that ROS production and intracellular Ca² ⁺ dysregulation interact in a feed-forward manner, thereby aggravating neuronal injury [4]. Although rotenone exposure does not reproduce all pathological features of PD, it provides a useful experimental model for studying mitochondrial stress-associated Ca² ⁺ dysregulation at the cellular level and has therefore been widely used to investigate oxidative stress-related neuronal injury.

Among voltage-gated calcium channels, L-type Ca^2+^ channels (LTCCs) are major determinants of activity-dependent Ca² ⁺ entry and Ca² ⁺ -mediated neuronal stress [2]. Dihydropyridine LTCC blockers, such as isradipine and nimodipine, have been explored experimentally for their potential preventive effect on PD; however, their clinical utility is limited by cardiovascular liabilities due to LTCC inhibition in cardiac and vascular smooth muscle [5]. For this reason, identifying complementary pharmacological targets and pathways involved in Ca² ⁺ regulation under oxidative stress conditions remains an important area of investigation. In this context, protein kinase A (PKA), which regulates LTCC-mediated Ca^2+^ influx [6], and phospholipase D (PLD), which is implicated in neuronal loss and inflammatory responses [7,8], have emerged as potential pharmacological targets associated with intracellular Ca² ⁺ regulation. Overexpression of PLD in the substantia nigra induces rapid neurodegeneration of dopaminergic neurons, and this is dependent on phosphatidic acid production [7]. Because phosphatidic acid serves as an important second messenger regulating intracellular Ca² ⁺ mobilization, PLD overexpression may contribute to Ca² ⁺ dysregulation during neurodegeneration [9].

Ginsenosides, the principal bioactive saponins of *Panax ginseng* (P. ginseng), exhibit antioxidant, anti-inflammatory, and neuroprotective properties in various experimental models, with accumulating evidence suggesting modulation of intracellular Ca² ⁺ homeostasis. Several experimental studies have reported that selected ginsenosides demonstrate relatively favorable cardiovascular safety profiles in experimental systems [10]. Among ginsenosides, protopanaxatriol (PPT)-type ginsenosides Rg1 and Rg2 have demonstrated neuroprotective and Ca² ⁺ -related actions in excitable tissues [11,12]. Protopanaxadiol (PPD)-type ginsenoside Rd also has been shown to influence Ca² ⁺ handling and cell survival pathways in ventricular myocytes [13,14] and to exert antioxidative and anti-inflammatory effects in dopaminergic neurons [15]. Previous structure-function analyses suggest that PPD-type ginsenosides may exhibit broader antioxidant and cytoprotective pharmacological properties compared with PPT-type compounds [16]. However, the comparative pharmacological profiles of structurally distinct ginsenosides in rotenone-associated intracellular Ca² ⁺ dysregulation have not been systematically investigated.

In the present study, we compared the effects of PPT-type ginsenosides (Rg1 and Rg2) and the PPD-type ginsenoside (Rd) on intracellular Ca² ⁺ dysregulation in SH-SY5Y neuronal and BV2 microglial cells exposed to rotenone. We particularly focused on comparing the pharmacological sensitivity of PLD-, LTCC-, and PKA-associated pathways regulating Ca² ⁺ influx under mitochondrial oxidative stress conditions. Rather than identifying direct molecular targets, the present study was designed to characterize differential pharmacological response profiles among structurally distinct ginsenosides and to provide pharmacological evidence for their distinct Ca² ⁺ -regulatory properties in neuronal and microglial cells.

## Methods

### Cell culture and treatments

Human neuroblastoma SH-SY5Y cells and murine microglial BV2 cells (ATCC, USA) were maintained in Dulbecco’s Modified Eagle Medium (DMEM) supplemented with 10% fetal bovine serum and 1% penicillin/streptomycin at 37℃ in a humidified atmosphere containing 5% CO_2_. SH-SY5Y and BV2 cells were used at passages 10−20. To induce mitochondrial stress relevant to rotenone-associated neurodegenerative conditions, cells were exposed to rotenone (10 nM) for 24 h, a concentration previously shown to induce oxidative stress and mitochondrial toxicity in neurons [17]. For comparative pharmacological analysis, cells were treated with ginsenosides Rg1, Rg2, and Rd (20 µM) immediately before the addition of rotenone (10 nM) [18]. Rotenone was then added directly into the same ginsenoside-containing medium without replacement, and cells were co-incubated with both compounds for 24 h in complete growth medium. All treatments in this study were performed in complete growth medium containing 10% FBS. After completion of the 24-h co-treatment period, cells were processed immediately for the respective assays. For intracellular Ca² ⁺ imaging, cells were washed with Hank’s Balanced Salt Solution (HBSS) and loaded with Fura-2 AM before fluorescence measurements. All ginsenosides and rotenone were dissolved in dimethyl sulfoxide (DMSO), and the final concentration of DMSO in the culture medium did not exceed 0.1% (v/v). Vehicle-treated control groups received an equivalent concentration of DMSO. This treatment protocol was designed to evaluate the protective pharmacological effects of ginsenosides during rotenone-induced cellular stress rather than their therapeutic effects after mitochondrial injury had already been established.

### Cell viability assay

To evaluate rotenone-induced cytotoxicity and determine an appropriate experimental concentration for subsequent Ca² ⁺ imaging experiments, SH-SY5Y and BV2 cells were divided into a vehicle-treated control group and rotenone-treated groups (0.1–10,000 nM). 5 × 10³ SH-SY5Y and BV2 cells were seeded in each well of a 96-well plate. For experiments assessing ginsenoside effects, cells were treated with Rg1, Rg2, and Rd (20 µM) immediately before rotenone exposure (10 nM) and co-incubated for 24 h, using the same treatment sequence as in Ca² ⁺ imaging experiments. After treatment, cells were incubated with 3-(4,5-dimethylthiazol-2-yl)-2,5-diphenyltetrazolium bromide (MTT) solution (0.5 mg/mL) for 1 h. Absorbance of DMSO-dissolved formazan crystals was read at 570 nm and 690 nm using a microplate reader (SPECTROstar Nano; BMG LABTECH, Germany). Cell viability was expressed as a percentage of the vehicle-treated control group. Each experiment was independently performed at least three times.

### Real-time cell confluence analysis

To assess rotenone-induced changes in cell growth and cytotoxic stress, SH-SY5Y and BV2 cells were assigned to vehicle-treated control and rotenone-treated groups. 1 × 10^4^ SH-SY5Y and BV2 cells were seeded in each well of 24-well plate. Real-time cell confluence was monitored using an automated live-cell imaging system (Celloger Mini Plus; Curiosis Inc., Republic of Korea). Cells were treated according to the experimental protocol described above, and confluence was quantified 24 h after exposure in rotenone-containing culture medium. This analysis was performed using the same treatment protocol used for the cell viability and Ca² ⁺ imaging experiments.

### Measurement of superoxide dismutase (SOD)

To evaluate intracellular antioxidant capacity under rotenone-induced oxidative stress, SOD activity was quantified using a commercially available colorimetric assay kit (EIASODC, Invitrogen, UK), according to the manufacturer’s protocol. 1 × 10^5^ SH-SY5Y and BV2 cells were seeded in each 60-mm culture dish. Cells were homogenized in cold PBS, and the supernatants were collected for analysis. SOD activity was determined based on inhibition of superoxide-driven tetrazolium reduction. Superoxide anions generated by the xanthine/xanthine oxidase reaction reduce tetrazolium salt to form formazan dye, and the rate of formazan formation is inversely proportional to SOD activity. Absorbance of supernatants was measured at 450 nm using a microplate reader, and SOD activity was normalized to total protein concentration.

To distinguish mitochondrial and cytosolic antioxidant capacity, total SOD activity was first measured under standard conditions. Mitochondrial SOD activity was selectively quantified in the presence of potassium cyanide (KCN), and cytosolic SOD activity was calculated by subtraction. Cells were treated using the same co-treatment protocol described for the Ca² ⁺ imaging experiments.

### Enzyme-linked immunosorbent assay

To validate rotenone-induced inflammatory activation, interleukin-6 (IL-6) levels in the culture supernatants were quantified using a commercial enzyme-linked immunosorbent assay (ELISA) kit (ab222503, R&D Systems, USA), according to the manufacturer’s protocol. 1 × 10^4^ SH-SY5Y and BV2 cells were seeded in each well of a 24-well plate and treated according to the same co-treatment protocol used for the Ca² ⁺ imaging experiments. Samples were added to the antibody-coated wells and incubated with detection reagents. After substrate addition and reaction termination, absorbance was measured at 450 nm using a microplate reader. The minimal detectable dose (sensitivity) of the assay was 11.3 pg/mL, with a dynamic range of 15.6–1000 pg/mL. IL-6 concentrations in SH-SY5Y cells that were below the assay detection limit were interpreted with caution.

### Measurement of intracellular Ca^2+^

Intracellular Ca² ⁺ dynamics were assessed using Fura-2 AM ratiometric fluorescence analysis. 5 × 10^5^ SH-SY5Y, BV2 cells were seeded in each 100-mm culture dish and as in the other experiments described above, rotenone and ginsenosides were co-administered in serum-containing medium for 24 h. After treatment, cells were detached from the dish using trypsin, and suspended in a standard extracellular solution containing 150 mM NaCl, 10 mM HEPES, 10 mM glucose, 6 mM KCl, 1.5 mM CaCl_2_, and 1 mM MgCl_2_ (pH 7.4, adjusted with NaOH). Cells (approximately 1 × 10⁶ cells/mL) were loaded with a ratiometric fluorescent indicator Fura-2 AM (2.5 µM) for 30 min. Store-operated Ca^2+^ entry (SOCE) was induced by depletion of endoplasmic reticulum Ca² ⁺ stores using 2’,5’-di(tert-butyl)-1,4-benzohydroquinone (BHQ, 30 µM), followed by re-addition of extracellular Ca^2+^ (1.5 mM), as previously described [19]. Intracellular Ca² ⁺ concentration ([Ca^2+^]ᵢ) was recorded using a fluorescence spectrometer (Photon Technology Ltd, USA) at 340/380 nm excitation and 510 nm emission. [Ca^2+^]ᵢ was calculated as [Ca^2+^]ᵢ = K_d_ × b × (R-R_min_)/(R_max_-R). K_d_ is the dissociation constant of Fura-2 for Ca^2+^ (224 nM), and b is the ratio of fluorescence intensity at 380 nm under Ca² ⁺ -free and Ca² ⁺ -saturated conditions. Quantitative analysis of Ca² ⁺ influx was performed by calculating the area under the curve (AUC) using Microcal Origin 6.0 software (Microcal software Inc., Northampton, USA).

To evaluate the pharmacological sensitivity of Ca² ⁺ influx responses, pharmacological inhibitors were applied during Ca² ⁺ measurements, including FIPI (PLD inhibitor, 500 nM), nifedipine (LTCC blocker, 10 µM), and H-89 (PKA inhibitor, 10 µM). Nifedipine and H-89 were also applied in combination to examine whether rotenone-associated Ca² ⁺ influx and ginsenoside-related Ca² ⁺ responses were associated with PKA-related modulation of LTCC activity. Inhibitors were applied during the Ca² ⁺ influx phase following extracellular Ca² ⁺ re-addition [20]. All experiments were performed in both SH-SY5Y neuronal cells and BV2 microglial cells.

### Malondialdehyde (MDA) assay

To assess rotenone-induced intracellular oxidative stress and the antioxidant effects of ginsenosides, MDA levels in cell homogenates were measured using a lipid peroxidation assay kit (ab118970, Abcam, USA) according to the manufacturer’s instructions. 1 × 10^5^ SH-SY5Y and BV2 cells were seeded in each well of a 6-well plate. Cell lysates were prepared and mixed with thiobarbituric acid (TBA) agent to form an MDA-TBA adduct, followed by incubation at 95°C for 60 min and cooling on ice. The reaction mixtures were transferred to a 96-well plate, and absorbance was recorded at 532 nm using a microplate reader at room temperature. The measured values were normalized to total protein concentration. The assay sensitivity (minimal detectable dose) was 0.1 nmol/well.

### Chemicals

Dulbecco’s Modified Eagle’s Medium (DMEM) and phosphate-buffered saline (PBS) were purchased from Welgene (Gyeongsan, Republic of Korea). Penicillin/streptomycin and fetal bovine serum (FBS) for culture media were purchased from Biowest (Riverside, MO, USA). The MTT reagent was purchased from Amresco (0793-1G, Solon, OH, USA), and Fura-2 acetoxymethyl ester (Fura-2 AM; F1221, purity > 95%) was obtained from Invitrogen (Paisley, UK). H-89 was purchased from Santa Cruz Biotechnology (Dallas, USA). All other chemicals, including rotenone, BHQ, ionomycin, FIPI, nifedipine, and ginsenosides Rg1, Rg2, and Rd were purchased from Sigma-Aldrich (St. Louis, USA). Fura-2 AM, rotenone, BHQ, ionomycin, ginsenosides Rg1, Rg2, and Rd were dissolved in DMSO, whereas other reagents were dissolved in sterile distilled water.

### Statistical analysis

Statistical analyses were conducted using IBM SPSS Statistics version 29 (Armonk, NY, USA). Data are presented as mean ± SEM. SEM was selected to describe the precision of the estimated group means under controlled experimental conditions, whereas statistical significance between groups was evaluated using one-way ANOVA followed by Tukey’s post hoc test. Student’s t-test was used for comparisons between two groups where appropriate. A p-value < 0.05 was considered statistically significant.

## Results

### Cellular responses to rotenone exposure in SH-SY5Y and BV2 cells

To evaluate cellular responses to rotenone exposure in SH-SY5Y and BV2 cells, cell viability, cell confluency, SOD activity, and IL-6 levels were measured. Cell viability and confluency assays were used to confirm rotenone-induced cytotoxic stress and to determine an appropriate experimental concentration for subsequent Ca² ⁺ imaging experiments. The MTT assay reflects mitochondrial metabolic activity, which serves as an indicator of cell viability, whereas cell confluency reflects the proportion of the culture surface area occupied by adherent cells. Therefore, these two assays evaluate different aspects of cellular status and may not produce identical quantitative changes.

**Fig 1:**
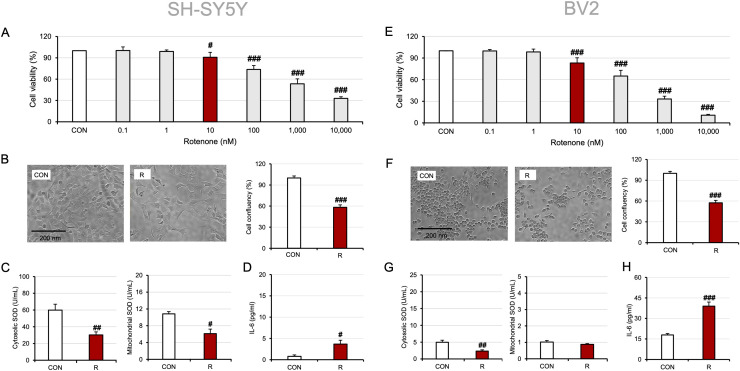
Rotenone (R)-induced cellular stress responses in SH-SY5Y and BV2 cells. (A, E) Cell viability assessed by MTT activity in SH-SY5Y (n = 6–7) and BV2 cells (n = 6–8). (B, F) Representative images and quantitative analysis of cell confluency in SH-SY5Y neuronal cells (n = 7) and BV2 microglial cells (n = 7). (C, G) Quantitative analysis of cytosolic and mitochondrial superoxide dismutase activity in SH-SY5Y (n = 6) and BV2 cells (n = 4–6). (D, H) Interleukin-6 (IL-6) levels in SH-SY5Y (n = 6) and BV2 cells (n = 10–12). Rotenone exposure induced cytotoxic, oxidative, and inflammatory stress responses under the experimental conditions used for Ca² ⁺ imaging experiments. Data are presented as mean ± SEM. Statistical analyses were performed using Student’s *t*-test or one-way ANOVA followed by Tukey’s post hoc test. ^#^p  < 0.05, ^##^p  < 0.01, ^###^p  <  0.001 vs. control (CON).

Rotenone exposure resulted in concentration-dependent decreases in MTT activity (Fig 1A, E). At 10 nM, rotenone significantly reduced cell viability and cell confluency in SH-SY5Y cells (p = 0.020, p < 0.001; Fig 1A, B) and BV2 cells (p < 0.001, respectively; Fig 1E, F). Although the quantitative changes differed because the two assays measure different biological parameters, both consistently demonstrated significant rotenone-induced cytotoxic stress in SH-SY5Y and BV2 cells. Based on these findings, 10 nM rotenone was used in subsequent experiments.

Cytosolic and mitochondrial SOD activities were measured to verify whether rotenone induces oxidative stress in neuronal and microglial cells. Rotenone significantly decreased cytosolic and mitochondrial SOD in SH-SY5Y cells (p = 0.004, p = 0.012; Fig 1C), and cytosolic SOD in BV2 cells (p = 0.004; Fig 1G). IL-6 levels were measured to further evaluate rotenone-associated inflammatory responses. Rotenone markedly increased IL-6 secretion in both cell types (p < 0.001 respectively; Fig 1D, H). However, because the IL-6 levels measured in SH-SY5Y cells were below the minimal detectable dose of the assay, these results should not be interpreted as evidence of neuronal inflammatory activation but rather as supportive data providing supportive evidence complementary to the intracellular Ca² ⁺ findings.

### Effects of ginsenosides on rotenone-induced Ca² ⁺ dysregulation

**Fig 2:**
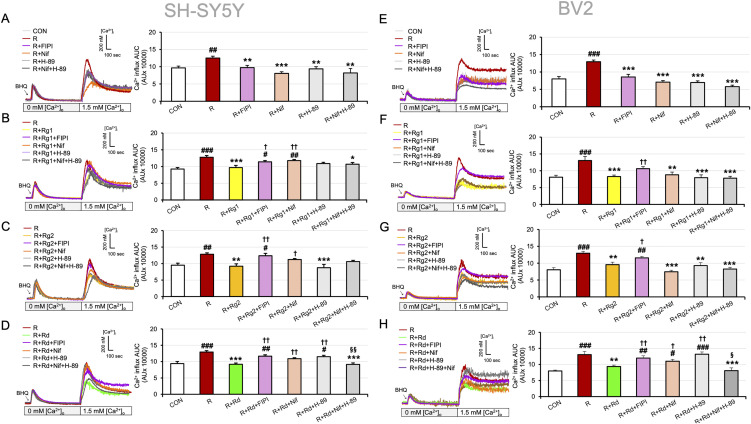
Pharmacological modulation of Ca² ⁺ influx–associated pathways in rotenone (R)-treated neuronal and microglial cells. (A, E) Representative intracellular Ca² ⁺ traces and corresponding AUC values following re-addition of extracellular Ca^2+^ (1.5 mM) after BHQ-mediated store depletion in SH-SY5Y (n = 9–11) and BV2 cells (n = 10–11). Cells were treated with rotenone (10 nM) in the presence or absence of inhibitors, including FIPI, nifedipine (Nif), and H-89. (B-D, F-H) Representative Ca² ⁺ traces and AUC values in SH-SY5Y (n = 9–11) and BV2 cells (n = 10–11) treated with ginsenosides immediately before rotenone exposure and acute inhibitor application during the Ca² ⁺ influx phase. For Rg2, SH-SY5Y (n = 9–10) and BV2 cells (n = 10–11) were analyzed, and for Rd, SH-SY5Y (n = 10–11) and BV2 cells (n = 9–11) were analyzed. Combined application of nifedipine and H-89 was used to assess PKA-associated modulation of LTCC-sensitive Ca² ⁺ responses. Data are presented as mean ± SEM. Statistical analyses were performed using Student’s *t*-test or one-way ANOVA followed by Tukey’s post hoc test. ^#^p  < 0.05, ^##^p  < 0.01, ^###^p  <  0.001 vs. control (CON); ^*^p < 0.05, ^**^p < 0.01, ^***^p < 0.001 vs. R; ^†^p < 0.05, ^††^p < 0.01 vs. ginsenosides-treated group; ^§^p < 0.05, ^§§^p < 0.01 vs. R+ginsenosides+Nif group.

To examine intracellular Ca² ⁺ responses under rotenone-induced stress conditions, SH-SY5Y and BV2 cells were exposed to rotenone (10 nM). Rotenone significantly increased Ca² ⁺ influx in both SH-SY5Y and BV2 cells (p = 0.007, p < 0.001; Fig 2A, E).

To distinguish endoplasmic reticulum Ca² ⁺ release from extracellular Ca² ⁺ influx, Ca² ⁺ responses were analyzed during BHQ-induced store depletion and following re-addition of extracellular Ca² ⁺ . Neither peak amplitude nor area under the curve (AUC) of BHQ-induced ER Ca² ⁺ release differed significantly among groups. Accordingly, quantitative analyses focused on extracellular Ca² ⁺ influx following Ca² ⁺ re-addition. The relative increase in Ca² ⁺ influx was greater in BV2 than SH-SY5Y cells (161% vs. 129%). Treatment with ginsenosides Rg1, Rg2, and Rd alone did not significantly affect basal Ca² ⁺ levels. However, co-treatment with ginsenosides significantly attenuated rotenone-induced Ca² ⁺ influx in both SH-SY5Y cells (p < 0.001, p = 0.003, and p < 0.001, respectively; Fig 2B-D) and BV2 cells (p < 0.001, p = 0.004, and p = 0.011, respectively; Fig 2F-H).

To explore pharmacological sensitivity of Ca² ⁺ influx responses, inhibitors targeting PLD (FIPI), LTCC (nifedipine), and protein kinase A (H-89) were applied during Ca² ⁺ measurements. Rotenone-induced Ca² ⁺ influx was significantly attenuated by FIPI, nifedipine, and H-89 in both SH-SY5Y cells (p = 0.004, p < 0.001, and p = 0.001, respectively; Fig 2A) and BV2 cells (p < 0.001 for all inhibitors; Fig 2E). These findings suggest pharmacological associations of rotenone-induced Ca² ⁺ influx with PLD-, LTCC-, and PKA-related components in both cell types.

The effects of Rg1 and Rg2 on Ca² ⁺ influx in SH-SY5Y cells were significantly reduced by FIPI and nifedipine (Fig 2B, C). In BV2 cells, only PLD inhibition significantly attenuated these effects (Fig 2F, G). H-89 did not significantly alter Ca² ⁺ responses in either cell type. These results indicate that the pharmacological responses to Rg1 and Rg2 were more closely associated with PLD-related components, with additional LTCC-sensitive responses in neuronal cells. In contrast, the effects of Rd were significantly reduced by FIPI, nifedipine, and H-89 in both SH-SY5Y cells and BV2 cells (Fig 2D, H). Combined treatment with nifedipine and H-89 produced greater suppression than nifedipine alone. These findings indicate that Rd exhibited broader pharmacological response profiles involving PLD-, LTCC-, and PKA-associated components.

Overall, all ginsenosides exhibited PLD-associated the pharmacological sensitivity, whereas Rg1 and Rg2 showed more restricted profiles and Rd exhibited broader pathway-associated responses. These findings characterize pharmacological response profiles inferred from inhibitor-based analyses and do not establish direct molecular mechanisms.

### Effects of ginsenosides on rotenone-induced increases in MDA

**Fig 3:**
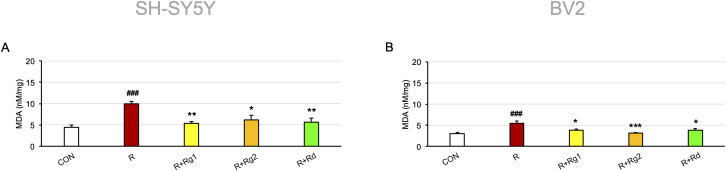
Effects of ginsenosides on rotenone (R)-treated neuronal and microglial cells. A, B) Bar graphs showing MDA levels in (A) SH-SY5Y and (B) BV2 cells. SH-SY5Y cells (n = 5) and BV2 cells (n = 5) were treated with ginsenosides Rg1, Rg2, and Rd (20 µM) immediately before rotenone (10 nM) administration and co-incubated for 24 h according to the experimental protocol described in the Methods section. Data are presented as mean ± SEM. Statistical analyses were performed using one-way ANOVA followed by Tukey’s post hoc test. ^###^p < 0.001 vs. control (CON); ^*^p < 0.05, ^**^p < 0.01, ^***^p < 0.001 vs. R.

To evaluate the effects of ginsenosides Rg1, Rg2, and Rd on rotenone-induced oxidative stress, we measured MDA levels in SH-SY5Y and BV2 cells. Rotenone (10 nM) significantly increased MDA levels in both SH-SY5Y and BV2 cells compared with control (p < 0.001, p < 0.001; Fig 3A, B). However, ginsenosides Rg1, Rg2, and Rd significantly reduced the rotenone-induced increase in MDA levels in both SH-SY5Y (p = 0.002, p = 0.010, and p = 0.003, respectively; Fig 3A) and BV2 cells (p = 0.017, p < 0.001, and p = 0.016, respectively; Fig 3B). These findings provide additional biochemical evidence that rotenone induces oxidative stress in both SH-SY5Y and BV2 cells and that ginsenosides Rg1, Rg2, and Rd significantly attenuate this oxidative stress.

## Discussion

In this study, rotenone exposure increased intracellular Ca² ⁺ influx in both neuronal and microglial cells. These responses were accompanied by reduced cell viability, decreased SOD activity, and increased IL-6 levels, indicating oxidative and inflammatory cellular stress under rotenone exposure conditions. Pharmacological inhibitor analyses howed that rotenone-associated Ca² ⁺ dysregulation was sensitive to PLD-, LTCC-, and PKA-related inhibition. Co-treatment with ginsenosides attenuated rotenone-induced Ca² ⁺ influx in both SH-SY5Y and BV2 cells. Among the compounds examined, Rg1 and Rg2 showed predominantly PLD-associated pharmacological responses, whereas Rd exhibited broader inhibitor-sensitive responses involving PLD-, LTCC-, and PKA-associated components. These ginsenosides also significantly attenuated the rotenone-induced increase in MDA levels in both SH-SY5Y and BV2 cells. Collectively, these findings indicate that ginsenosides attenuate rotenone-associated intracellular Ca² ⁺ dysregulation and exert antioxidant effects under oxidative stress conditions.

**Fig 4:**
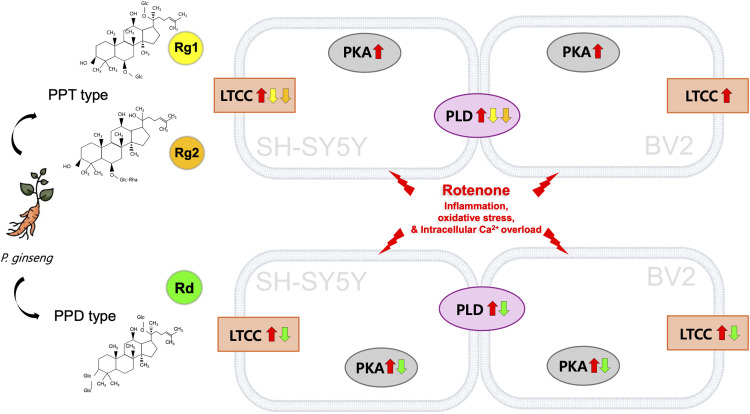
Schematic summary of ginsenoside-associated Ca² ⁺ responses in rotenone-treated SH-SY5Y and BV2 cells. Schematic illustration summarizing the differential responses of ginsenosides Rg1, Rg2, and Rd in rotenone-associated Ca² ⁺ dysregulation in neuronal (SH-SY5Y) and microglial (BV2) cells. The schematic was generated based on inhibitor-sensitive response patterns observed in the present study and does not represent direct molecular mechanisms. Rg1 and Rg2 showed predominantly PLD-associated responses, with additional LTCC-sensitive components in neuronal cells. In contrast, Rd showed broader pharmacological sensitivity involving PLD-, L-type Ca^2+^ channel (LTCC)-, and protein kinase A (PKA)-associated components in both cell types. Red arrows indicate pathways pharmacologically associated with rotenone-induced cellular stress and Ca² ⁺ dysregulation, whereas green and yellow lines indicate ginsenoside-associated modulation of Ca² ⁺ influx responses. Abbreviations: P. ginseng, *Panax ginseng*; PPT, Protopanaxatriol; PPD, Protopanaxadiol; LTCC, L-type Ca² ⁺ channel; PKA, protein kinase A; PLD, phospholipase D.

Fig 4 summarizes the pharmacological response patterns observed in this study. The schematic was intended to summarize inhibitor-sensitive responses under rotenone-induced stress conditions and was not designed to define direct molecular targets or signaling mechanisms. Rg1, Rg2, and Rd all showed sensitivity to PLD inhibition, suggesting that PLD-associated components contribute to Ca² ⁺ regulation in both neuronal and microglial cells. PLD isoforms are involved in vesicular trafficking and lipid-associated signaling pathways, and dysregulation of these pathways has been linked to inflammatory and neurodegenerative processes in the nervous system [21]. The present findings are consistent with previous reports suggesting involvement of PLD-associated signaling during cellular stress conditions such as oxidative stress [22]. To our knowledge, this study provides the first pharmacological evidence suggesting that ginsenosides are associated with PLD-related regulation of excessive Ca² ⁺ influx under rotenone-induced stress conditions. Based on these findings, further studies are needed to clarify the molecular mechanisms by which ginsenosides modulate PLD-associated responses, which may provide additional insight into their potential therapeutic relevance in neurodegenerative diseases including Parkinson’s disease.

Ginsenosides can be broadly classified into PPT- and PPD-type compounds, and previous studies have shown that structural differences among ginsenosides influence membrane interaction and pharmacological activity [23,24]. In the present study, Rg1 and Rg2 showed more pronounced cell type-dependent pharmacological responses than Rd. In SH-SY5Y cells, their effects were sensitive to both LTCC- and PLD-related inhibition, whereas in BV2 cells, PLD-associated pharmacological responses predominated. These findings suggest that neuronal and microglial cells differ in pharmacological responses associated with Ca² ⁺ influx regulation under rotenone-induced stress conditions.

Rd-associated Ca² ⁺ responses were sensitive to both kinase-related and LTCC-related inhibitors, suggesting the involvement of multiple Ca² ⁺ influx-associated components. In neurons, LTCC-mediated Ca² ⁺ influx is regulated in part by phosphorylation-dependent pathways involving PKA [6]. Previous studies have also suggested associations between Rd and cAMP/PKA-related signaling pathways. To our knowledge, this study provides the first pharmacological evidence that Rd-associated Ca² ⁺ responses involve PKA/LTCC-sensitive components under rotenone-induced stress conditions. Although the present experiments were not designed to identify direct molecular interactions through PKA/LTCC-associated pharmacological responses, our results provide pharmacological evidence suggesting the involvement of PKA in ginsenoside Rd-mediated regulation of LTCC-mediated Ca² ⁺ influx. Furthermore, our findings suggest that PKA/LTCC-associated signaling may represent a potential therapeutic target for intracellular Ca² ⁺ imbalance associated with neurodegenerative diseases, including Parkinson’s disease.

Several limitations should be considered. First, although SOD activity and IL-6 levels were measured to confirm rotenone-induced stress responses, the effects of ginsenosides on these markers were not systematically evaluated. In addition, the IL-6 levels measured in SH-SY5Y cells were below the minimal detectable dose, which limited the interpretation that rotenone induced an inflammatory response. However, previous studies have reported that rotenone increases intracellular Ca² ⁺ levels while simultaneously decreasing SOD activity [25]. In addition, rotenone activates NMDARs to increase Ca² ⁺ influx, and elevated IL-6 levels have also been shown to activate NMDARs [26,27]. Collectively, these findings suggest that oxidative stress and inflammatory signaling may cooperatively contribute to rotenone-associated intracellular Ca² ⁺ dysregulation. Second, the present study used pharmacological inhibitor-based analyses without direct biochemical assessment of PLD isoforms, PKA activity, or LTCC regulation. Additional molecular and genetic approaches will be necessary to further define the mechanisms associated with ginsenoside-mediated Ca² ⁺ regulation under rotenone-induced oxidative stress conditions. Third, this study has a limitation that it is based on cell-based experimental results, which do not fully mimic the physiological environment of the human body. Additionally, our findings reflect both cell type-specific and potential species-specific responses. Therefore, caution is warranted when extrapolating these findings to human Parkinson’s disease. Fourth, although rotenone may affect intracellular Ca^2+^ through ATP depletion, this present study was conducted without direct assessment of intracellular ATP levels. Further studies incorporating direct ATP measurements and mitochondrial function-enhancing agents, such as coenzyme Q10, will help clarify this possibility.

## Conclusion

This study showed that ginsenosides differentially affected Ca² ⁺ influx-associated responses under rotenone-induced mitochondrial stress conditions. PPT-type ginsenosides Rg1 and Rg2 showed predominantly PLD-associated pharmacological responses in both neuronal and microglial cells, with additional LTCC-sensitive responses in neuronal cells. In contrast, the PPD-type ginsenoside Rd showed broader pharmacological sensitivity involving PLD-, LTCC-, and PKA-associated components. The consistent sensitivity of Ca² ⁺ responses to PLD inhibition in both cell types suggests that PLD-associated pharmacological pathways contribute to rotenone-associated Ca² ⁺ dysregulation. In addition, ginsenosides significantly attenuated rotenone-induced lipid peroxidation, as demonstrated by reduced MDA levels in both neuronal and microglial cells. Overall, these findings provide pharmacological evidence that ginsenosides differentially modulate Ca² ⁺ -related pharmacological responses and oxidative stress under rotenone-induced mitochondrial stress conditions.

## Supporting information

S1 Data Rawdata. (XLSX)
