# Supplementary material for: Differential effects of ginsenosides on Ca2+ regulation in rotenone-treated neuronal and microglial cells
Source: PLoS One. 2026 Aug 18;21(8):e0356602. doi: 10.1371/journal.pone.0356602 (PMC13484979; doi:10.1371/journal.pone.0356602)
Supplement: Supplementary file 1 [file pone.0356602.s002.docx]

**Response to the Editor**

We sincerely thank the Editor for the careful evaluation of our manuscript and for providing constructive comments. We greatly appreciate the opportunity to revise our manuscript. Following the Editor's recommendations and the detailed comments from the four reviewers, we have substantially revised the manuscript to improve its clarity, methodological transparency, and scientific rigor. **In response to the reviewers' major concerns, we not only extensively revised the manuscript but also performed additional experiments measuring malondialdehyde (MDA), a widely accepted marker of lipid peroxidation, to provide** additional biochemical evidence of oxidative stress under rotenone treatment. **These new experimental data further strengthen the biological basis of our conclusions and have been incorporated into the revised manuscript as a new main figure.** All revisions are highlighted in the revised manuscript, and detailed point-by-point responses to each reviewer comment are provided below.

## **Journal Requirement #2**

**Comment**

Please state what role the funders took in the study. If the funders had no role, please state: ‘The funders had no role in study design, data collection and analysis, decision to publish, or preparation of the manuscript.’

**Response:**

**Thank you for this instruction. We have revised the Role of the Funder statement as requested. The revised Funding Statement now reads:**

"This work was supported by the National Research Foundation (NRF) of Korea grant funded by the Korea government (MSIT) (RS-2024-00353184) and the Institute of Nursing Research, Korea University Grant. The funders had no role in study design, data collection and analysis, decision to publish, or preparation of the manuscript."

This statement has also been included in the Cover Letter so that the Funding Statement in the online submission system can be updated accordingly.

## **Journal Requirement #3**

**Comment**

Funding information should not appear in the Acknowledgments section. Please update the Funding Statement accordingly.

**Response:**

**Funding-related text has been removed from the Acknowledgments section in accordance with the Journal Requirements, and the revised Funding Statement has been provided in the Cover Letter for updating the online submission system.**

**Additional Editor Comments:**

After careful evaluation, including comments from four independent reviewers, I believe that the manuscript addresses an interesting topic and presents potentially valuable findings regarding the effects of ginsenosides on calcium regulation under rotenone-induced cellular stress. However, several important issues must be addressed before the manuscript can be considered further for publication.

The reviewers identified a number of concerns that require substantial revision. In particular, the revised manuscript should:

• Provide more detailed methodological information to ensure reproducibility, including experimental design, cell culture conditions, cell seeding densities, treatment procedures, assay conditions, normalization approaches, and replication details.

**Response:**
We sincerely thank the Editor for the careful evaluation of our manuscript and for providing constructive comments. We greatly appreciate the opportunity to revise our manuscript. Following the Editor's recommendations and the detailed comments from the four reviewers, we have substantially revised the manuscript to improve its clarity, methodological transparency, and scientific rigor. **In response to the reviewers' major concerns, we not only extensively revised the manuscript but also performed additional experiments measuring malondialdehyde (MDA), a widely accepted marker of lipid peroxidation, to provide** additional biochemical evidence of oxidative stress under rotenone treatment. These new experimental data further strengthen the biological basis of our conclusions and have been incorporated into the revised manuscript as a new main figure together with the corresponding Results and Discussion.

All revisions are highlighted in the revised manuscript, and detailed point-by-point responses to each reviewer comment are provided below.

• Clarify the experimental workflow, particularly the pretreatment design and the interpretation of the calcium measurements and inflammatory marker analyses.

**Response:**
Thank you for this helpful comment. We have clarified the overall experimental workflow throughout the Methods and Results sections. The treatment protocol has been described more explicitly, including the timing of ginsenoside administration, the continuous presence of ginsenosides during rotenone exposure, and clarification that the experimental design reflects a co-treatment protocol rather than a distinct pretreatment phase. The calcium imaging protocol has also been clarified, including the distinction between ER Ca²⁺ release and extracellular Ca²⁺ influx, the rationale for AUC analysis, and normalization procedures. In addition, the rationale for measuring SOD activity and IL-6 as indicators of oxidative and inflammatory stress has been expanded, and the interpretation of these findings has been clarified in both the Results and Discussion sections. To improve transparency, the experimental timeline and treatment sequence have been revised to accurately describe the co-treatment protocol used throughout the study. **We also clarified the rationale for selecting the rotenone treatment conditions used for the subsequent Ca²⁺ imaging experiments.**

• Strengthen the discussion of the proposed mechanisms involving calcium regulation, mitochondrial dysfunction, oxidative stress, and the potential roles of PLD- and PKA-related pathways, while avoiding conclusions that extend beyond the data presented.

**Response:**
We agree with the Editor's recommendation. The Discussion has been extensively revised to better integrate our findings with previous literature regarding mitochondrial dysfunction, oxidative stress, intracellular Ca²⁺ regulation, and the potential involvement of PLD- and PKA-associated signaling pathways. To further strengthen the evidence for oxidative stress, we performed additional experiments measuring malondialdehyde (MDA), and these new data have been incorporated into the revised Results and Discussion sections. **A new figure presenting the MDA results has been added to the revised manuscript to provide independent biochemical evidence supporting rotenone-induced oxidative stress and its attenuation by ginsenosides.** We also revised several statements throughout the manuscript to avoid overinterpretation and to emphasize that our findings demonstrate pharmacological response profiles and pharmacological associations rather than direct molecular mechanisms. Furthermore, the Discussion now more clearly distinguishes experimentally supported findings from hypotheses that require future mechanistic investigation.

• Address the reviewers’ concerns regarding statistical reporting, validation of key assays, biological significance of the observed effects, and the translational relevance of the findings.

**Response:**
Thank you for this valuable suggestion. We have addressed all reviewer concerns regarding statistical analyses by providing additional methodological details, clarifying sample sizes and statistical procedures, and improving the presentation of the results. Additional information regarding assay validation, including assay sensitivity, dynamic range, and methodological limitations where appropriate. The Discussion has also been expanded to better explain the biological significance of the findings, their potential translational relevance, and the limitations of the present in vitro study, including issues related to blood-brain barrier permeability, pharmacokinetics, and future in vivo validation. Where appropriate, we also incorporated additional references suggested by the reviewers and revised the interpretation of our findings to better align with the scope of the experimental data. **Collectively, these revisions improve both the methodological rigor and the biological interpretation of the study while maintaining conclusions that are fully supported by the experimental data.**

Please provide a detailed point-by-point response to all reviewer comments and clearly indicate all changes made in the revised manuscript.

In view of the extent of the revisions required, I am inviting you to submit a revised version of your manuscript within 45 days.

I look forward to receiving your revised submission.

Once again, we sincerely thank the Editor and reviewers for their thoughtful comments, which have substantially improved the quality and clarity of our manuscript. We believe that the revised manuscript has been substantially strengthened through additional experimental validation, expanded methodological descriptions, improved transparency, and a more balanced interpretation of the findings. We have carefully addressed every comment raised by the Editor and reviewers in a point-by-point manner and have clearly indicated all revisions in the manuscript. We hope that the revised manuscript satisfactorily addresses all concerns and will now be suitable for further consideration for publication in PLOS ONE.

**Review Comments to the Author**

Please use the space provided to explain your answers to the questions above. You may also include additional comments for the author, including concerns about dual publication, research ethics, or publication ethics. (Please upload your review as an attachment if it exceeds 20,000 characters)

**Reviewer #1:**

In this study, the authors tried to demonstrate the neuroprotective effects of ginsenosides against rotenone-induced mitochondrial toxicity. They proposed that PPT-type ginsenosides Rg1 and Rg2 mainly activate PLD-related signaling in both neuronal and microglial cells and also exhibit responses sensitive to LTCC in neurons. On the other hand, the PPD-type ginsenoside Rd demonstrated a broader range of pharmacological sensitivity, engaging PLD, LTCC, and PKA pathways. The persistent Ca²⁺ sensitivity to PLD inhibition in both cell types suggests that PLD-related pathways are involved in rotenone-induced Ca²⁺ dysregulation. This reviewer’s points are as follows:

1. This study requires minor paraphrasing and proofreading.

### Response:

Thank you for your insightful comments. We have carefully revised the manuscript through paraphrasing and proofreading to improve its overall quality. **In addition, we revised the title, Abstract, Introduction, Discussion, and figure legends to improve clarity, consistency in terminology, and scientific precision throughout the manuscript. Several statements were also rephrased to avoid overinterpretation and to ensure that the conclusions are fully supported by the experimental data.**

2. The present study seems like a short communication; the proper mechanistic overview is missing. The effect of ginsenoside on calcium homeostasis has been well demonstrated in mitochondrial toxicity assays, both in vitro and in vivo. However, the roles of PLD and PKA are novel. Therefore, they should highlight their novelty more strongly. Additionally, include more mechanistic data to establish a strong link between PLD and PKA-associated protective mechanisms.

**Response:**

Thank you for recognizing the novelty of our work. We specifically focused on phospholipase D (PLD) and protein kinase A (PKA) to evaluate the pharmacological sensitivity of ginsenosides. Previous studies have implicated PLD in neuronal degeneration and inflammatory responses in neurodegenerative diseases, and its product phosphatidic acid (PA) functions as a second messenger for Ca²⁺ mobilization (Siddiqui and English, 1997; Mendez-Gomez et al., 2018; Ke et al., 2024). PKA regulates L-type Ca²⁺ channel (LTCC)-mediated Ca²⁺ influx (Murphy et al., 2014). Based on these findings, we investigated whether ginsenosides could modulate rotenone-induced intracellular Ca²⁺ dysregulation through PLD- and PKA-associated pharmacological pathways.

We agree that direct mechanistic evidence would further strengthen our conclusions. However, the primary objective of the present study was to compare the pharmacological response profiles of structurally distinct ginsenosides using selective pharmacological inhibitors rather than to identify direct molecular targets. Accordingly, throughout the revised manuscript, we consistently describe these findings as pharmacological response profiles inferred from inhibitor-based analyses rather than direct molecular mechanisms.

In addition, to strengthen the biological relevance of the rotenone model, we performed new experiments measuring malondialdehyde (MDA), a marker of lipid peroxidation, and incorporated these data into the revised manuscript. The new MDA results complement the SOD findings and provide independent biochemical evidence supporting rotenone-induced oxidative stress.

Accordingly, we revised the Introduction and Discussion to better highlight the novelty of the pharmacological findings while clearly acknowledging the limitations of the current inhibitor-based approach.

**Reviewer #2:**

The manuscript entitled "Differential effects of ginsenosides on Ca²⁺ regulation in rotenone-treated neuronal and microglial cells" presents interesting findings regarding the effects of ginsenosides on calcium homeostasis under rotenone-induced stress. However, substantial revisions are required to improve methodological transparency and to strengthen the validation of both the study concept and the reported results.

1. My primary concern is the lack of sufficient methodological detail, which currently limits reproducibility and makes it difficult for readers to critically evaluate the findings. Several essential experimental parameters are missing or insufficiently described, including:
The number of cells seeded per well for each experimental assay.
Whether experiments were performed in complete growth medium or under serum-free conditions. This is particularly important given the 24-hour pretreatment period followed by an additional 24-hour treatment period as understood from the MS.
Whether the culture medium was replaced after the pretreatment phase or whether ginsenosides remained present during the subsequent rotenone exposure. Clarification is needed to determine whether the effects reflect pretreatment alone or co-treatment with rotenone.

**Response:**

We sincerely thank you for the thoughtful and constructive review. We agree that additional methodological detail is necessary to ensure reproducibility. Accordingly, we have substantially revised the methods section. We have also clarified the experimental protocol and calcium measurement procedure to improve methodological transparency and reproducibility.

All treatments in our experiments were conducted **in complete growth medium containing 10% fetal bovine serum (FBS),** and we added this information **to the Methods section.**

**Regarding the treatment protocol,** ginsenosides Rg1, Rg2, or Rd were added to the culture medium **immediately before** rotenone administration. Rotenone was then added directly into the same ginsenoside-containing medium without replacement, and cells were incubated with both compounds for 24 h. **Therefore, the observed effects reflect a co-treatment protocol rather than a distinct pretreatment phase.** We apologize for the earlier description implying a **24-h pretreatment followed by an additional 24-h treatment period,** and we have revised the Methods section **to accurately reflect the actual experimental protocol.** All corresponding descriptions in the Methods, Results, figure legends, and Discussion have been revised to ensure consistency with the actual experimental protocol.

For comparative pharmacological analysis, cells were **treated with** ginsenosides Rg1, Rg2, and Rd (20 µM) **immediately before** rotenone (10 nM) administration. Rotenone was then added directly into the same ginsenoside-containing medium without replacement, and cells were co-incubated with both compounds for 24 h in complete growth medium.

We have also added the number of cells seeded for each experimental assay in the Methods section.

2. The calcium measurement protocol requires considerably more detail. Specifically, the authors should explain how intracellular Ca²⁺ levels were measured following 24 hours of treatment and a total of 48 hours of compound exposure. Since prolonged exposure may affect cell viability and cell number, it is unclear how the measured fluorescence signals can be attributed to specific changes in calcium regulation rather than differences in viable cell density. Information regarding assay normalization and controls is necessary.

**Response:**

We thank you for pointing out this important issue. First, as noted above, ginsenosides and rotenone were co-administered for a total of 24 h. Regarding normalization, intracellular Ca²⁺ levels were measured using Fura-2 AM, a ratiometric fluorescent indicator. The 340/380 nm ratio was used to determine relative intracellular Ca²⁺ concentration. **Because Fura-2 is a ratiometric indicator, this ratio minimizes the influence of differences in cell number and dye loading efficiency.** We have added details of the Ca²⁺ measurement protocol and **data analysis.**

[Ca²⁺]ᵢ was calculated as [Ca²⁺]ᵢ = K_d_ × b × (R−Rmin)/(Rmax−R). K_d_ **is the dissociation constant of Fura-2 for Ca²⁺ (224 nM),** and b is the ratio of the maximum to minimum fluorescence intensity at 380 nm.

Untreated control cells were analyzed under the same experimental conditions, and all Ca²⁺ measurements were normalized using the Fura-2 ratiometric method.

3. In the Results section, Figure 1 raises several questions. If the red bars represent the same treatment concentration across all panels, the authors should explain why, in panels B and F, cell confluency decreases to approximately 60%, whereas cell viability remains between 80–90%. The apparent discrepancy between these measurements requires clarification.

**Response:**

Cell confluency and MTT results can differ because they measure different aspects of cells. Confluency reflects how much of the culture surface is covered, whereas the MTT assay reflects mitochondrial metabolic activity. Therefore, cells with reduced attachment or proliferation may still retain measurable metabolic activity, resulting in relatively preserved MTT values despite reduced confluency.

A clarifying statement has been added to the Results section to explain this methodological difference.

4. The IL-6 measurements also require further validation and discussion. The authors should provide:
The detection limit, dynamic range, and sensitivity of the assay kit used. Information on positive and negative controls included in the assay. Justification for the biological significance of the observed IL-6 changes, particularly given that the reported concentrations appear very low (approximately 5 pg/mL) and the maximal induction is only about two-fold.

**Response:**

The minimal detectable dose (sensitivity) of the assay used in this study is **11.3 pg/mL. Therefore,** the IL-6 levels observed in SH-SY5Y cells **were below** the detection limit.

We have added a statement that the results obtained from SH-SY5Y cells should be interpreted with caution, and that the IL-6 results were presented as **supportive evidence** of the inflammatory response to complement the Ca²⁺ findings.

The dynamic range of the assay was 15.6–1000 pg/mL, indicating that the BV2 IL-6 measurements were within the reliable quantification range.

IL-6 measurements were performed according to the manufacturer's instructions using the supplied calibration standards.

5. Overall, the study addresses an interesting topic; however, the current lack of methodological detail and insufficient validation of several key endpoints make it difficult to assess the robustness of the findings. Additional experimental details, clarification of the study design, and stronger justification of the biological significance of the reported effects are necessary before the conclusions can be fully evaluated.

**Response:**

We greatly appreciate your insightful comments. Your suggestions substantially improved the methodological transparency, reproducibility, and overall quality of the revised manuscript. We sincerely thank you for your careful review and valuable recommendations.

**Reviewer #3:** 
This is an interesting paper that studies the effect of ginsenosides on Ca²⁺ regulation in rotenone-treated neurons/microglia cells. However, I have some comments for the authors.

1. Since you are trying to mimic Parkinson`s disease did you ensure that complex I was inhibited as you only treated the cells with rotenone and didn`t assay complex I activity?

**Response:**

We deeply appreciate your insightful comments on our manuscript. This study was conducted to evaluate the pharmacological response of ginsenosides in neuronal and microglial cells exposed to rotenone. For this purpose, we used a rotenone concentration that **has previously been demonstrated** to inhibit mitochondrial complex I activity. Rotenone at 10 nM has been reported to inhibit complex I activity and induce oxidative **stress** in human neuroblastoma cells (Sherer et al., 2003). **Accordingly, rotenone was used as a well-established model of mitochondrial dysfunction rather than as a variable requiring independent validation in the present study.** Therefore, this study focused on **the downstream changes in intracellular Ca²⁺ regulation under rotenone-induced mitochondrial stress** rather than directly measuring complex I activity. **We have also acknowledged in the revised Discussion that direct measurement of complex I activity would further strengthen the mechanistic interpretation and should be addressed in future studies.**

2. I think more than one cell viability assay ids required in view of the limitations of the MTT assay. Did the authors assess any interference of the ginsenosides on the MTT assay. The full name of MTT is required before it is abbreviated as MTT.

**Response:**

We agree that additional experiments beyond the MTT assay are necessary to more comprehensively evaluate the effects of rotenone on neuronal and microglial cell viability. Therefore, we additionally assessed **cell confluency as an independent indicator of cell status,** thereby complementing the limitations of the MTT assay. As a result, both the MTT assay and cell confluency measurements showed significant reductions following treatment with 10 nM rotenone. **Because these two assays evaluate different biological parameters (metabolic activity versus adherent cell coverage), the observed differences between them are discussed in the revised manuscript.** In addition, **to further characterize rotenone-induced cellular stress, we evaluated oxidative stress (SOD activity and newly added malondialdehyde [MDA] measurements) and inflammatory responses (IL-6).** We also added the full name, **3-(4,5-dimethylthiazol-2-yl)-2,5-diphenyltetrazolium bromide (MTT),** at its first appearance in the manuscript (Page 5).

3. I couldn`t find any evidence increased reactive oxygen species or oxidative stress measured in the paper or SOD levels and inflammatory markers. The authors should measure markers of oxidative stress such as Malondialdehyde or levels of cellular/mitochondrial oxidative stress.

**Response:**

As you pointed out, we have **strengthened the evidence for oxidative stress** by additionally measuring **malondialdehyde (MDA), a well-established marker of lipid peroxidation**. The additional experiments demonstrated that rotenone significantly increased MDA levels in both SH-SY5Y neuronal cells and BV2 microglial cells, whereas co-treatment with ginsenosides significantly attenuated these increases. **These new data have been incorporated into the revised manuscript as a new main figure and complement the SOD findings, providing independent biochemical evidence that rotenone-induced Ca²⁺ dysregulation occurs in the context of oxidative stress.**

4. Do the ginsenosides pass through the blood brain barrier as if not this would be a limitation in there use as a therapeutic agent?

**Response:**

We agree that blood-brain barrier (BBB) permeability is an important consideration when discussing the therapeutic potential of ginsenosides. However, as the present study aimed to investigate the pharmacological effects of ginsenosides in cultured neuronal and microglial cells, BBB permeability was beyond the scope of this investigation. Previous studies have shown that brain exposure varies depending on the ginsenoside subtype, dose, formulation, and route of administration (Won et al., 2019). Although several studies have reported neuroprotective effects of ginsenosides in the central nervous system (Ong et al., 2015), our findings should be interpreted as evidence of cellular pharmacological responses rather than direct evidence of therapeutic efficacy in Parkinson's disease. We have therefore revised the Discussion to acknowledge this limitation and to state that further pharmacokinetic and in vivo studies are required before these findings can be translated into therapeutic applications.

Page 18:

Third, this study has a limitation that it is based on cell-based experimental results, which do not fully mimic the physiological environment of the human body. Furthermore, the blood-brain barrier permeability and pharmacokinetic properties of individual ginsenosides were not evaluated and should be investigated in future in vivo studies.

5. Surely the loss of ATP in the rotenone treated cells would effect calcium homoeostasis and therefore in addition to the use of ginsenosides other agents that improve mitochondrial function such as coenzyme Q10 should be considered as dual treatments?

**Response:**

We appreciate this thoughtful suggestion. The primary objective of the present study was to compare the pharmacological response profiles of different ginsenosides in regulating intracellular Ca²⁺ homeostasis under rotenone-induced stress. We agree that ATP depletion secondary to mitochondrial dysfunction may contribute to intracellular Ca²⁺ dysregulation and that combining ginsenosides with mitochondrial function-enhancing agents, such as coenzyme Q10, represents an interesting therapeutic strategy. Accordingly, we have expanded the Discussion to highlight combination therapy targeting both mitochondrial function and Ca²⁺ homeostasis as a promising direction for future investigation.

Page 19:

Fourth, although rotenone may affect intracellular Ca²⁺ homeostasis through ATP depletion, ATP levels were not directly evaluated in the present study. Further studies are needed to clarify this relationship and to determine whether combination treatment with mitochondrial function enhancers, such as coenzyme Q10, provides additional protective effects against rotenone-induced cellular stress.

6. On a minor point, the abstract requires an introductory sentences to provide the relevance of the study.

**Response:**

As you suggested, we revised the Abstract by adding an introductory sentence to better explain the scientific background and clinical relevance of the study before presenting the study objective and experimental approach. **The revised Abstract now more clearly introduces the relationship between mitochondrial dysfunction, oxidative stress, and intracellular Ca²⁺ dysregulation, thereby providing a stronger rationale for the present study.** We sincerely thank you for your thoughtful comments, which have greatly improved the quality and clarity of our manuscript.

**Reviewer #4:** 
Manuscript entitled „Differential effects of ginsenosides on Ca²⁺ regulation in rotenone-treated neuronal and microglial cells“ evaluated the effects of three structurally different ginsenosides on oxidative stress conditions induced by inhibitor of mitochondrial complex I.
Study showed new information about pharmacological activity of ginsenoside subtypes in rotenone-associated Ca²⁺ dysregulation in human neuronal SH-SY5Y cells and murine microglial BV2cell lines.

Comments:

1/ Justify why cell lines of different biological origin, i.e. human and mouse, were chosen. Could the observed differences in the effect of ginsenosides Rg1, Rg2, and Rd be related to the different biological origin of the cell lines?

**Response:**

We are grateful for your thoughtful review. The primary objective of this study was to examine the pharmacological responses of ginsenosides in neurons and microglia exposed to rotenone. In this context, we used SH-SY5Y and BV2 cells as experimental models representing neurons and microglia. Also, BV2 cells are often used in combination with human neuronal cell lines to investigate neuroimmune responses relevant to the human central nervous system (Schmidt et al., 2021). We acknowledge that species differences may have contributed to the observed responses. Therefore, the findings should be interpreted as reflecting both cell type-specific and potential species-specific differences. However, the differences observed in this study reflect both cell type-specific and potential species-specific responses. We have addressed both of these points in the discussion section.

Page 19:

Additionally, since our results were obtained using different biological origin cell lines, they encompass both cell type-specific and potential species-specific responses. Therefore, caution is warranted when interpreting the cell type-specific findings.

2/ The abbreviations for ginsenosides appeared for the first time in the Abstract, but the full names of the individual compounds were missing. This needs to be added.

**Response:**

Thanks to your insightful review, we revised the manuscript by adding all missing full names of abbreviations.

3/ Individual methodological procedures are described very briefly and lack information necessary for the possibility of reproducing in vitro experiments, for example, the number of cells used in individual tests and subsequent isolation from plastic.

**Response:**

We agree that methodological detail is needed for the reproducibility of the experiments. Accordingly, we have substantially revised our methods section. Details such as the number of seeded cells for each experiment have been added.

Page 5

Cell viability assay : 5 × 10³ SH-SY5Y and BV2 cells were seeded in each well of 96-well plate.

Page 6

Real-time cell confluency analysis: 1 × 10^4^ SH-SY5Y and BV2 cells were seeded in each well of 24-well plate.

Malondialdehyde (MDA) assay : 1 × 10^5^ SH-SY5Y and BV2 cells were seeded in each well of 6-well plate.

Measurement of Superoxide dismutase (SOD) : 1 × 10^5^ SH-SY5Y and BV2 cells were seeded in each 60-mm culture dish.

Page 7

Enzyme-linked immunosorbent assay: 1 × 10^4^ SH-SY5Y and BV2 cells were seeded in each well of 24-well plate.

Measurement of intracellular Ca^2+^ : 5 × 10^5^ SH-SY5Y, BV2 cells were seeded in each 100-mm culture dish.

We also expanded the description of the calcium measurement protocol and treatment procedure to further improve reproducibility.

4/ The authors used different numbers of replicates for each test and expressed results as mean ± SEM. Explain why the mean and SD, which are more statistically sensitive indicators of variability, were not calculated. Can you change calculations?

**Response:**

We appreciate the reviewer's thoughtful comment regarding the presentation of variability. We understand that our results may raise concerns from a statistical perspective. SD is a descriptive statistic that estimates how widely individual observations are scattered around the sample mean and is therefore more suitable for characterizing the sample itself. In contrast, we used the SEM which reflects how close the sample mean is likely to be to the true population mean and thus serves as an inferential statistic describing the error of the sample estimates. As our experiments were performed using a cell line under highly controlled conditions, in which biological inter-individual variability is minimal. Moreover, the primary aim of this study is not to determine the true mean in each treatment group, but rather to test for differences between groups. Accordingly, SEM was selected to present the precision of the estimated group means, whereas statistical significance between groups was evaluated using one-way ANOVA followed by Tukey's post hoc test (Lee et al., 2015)

5/ The study essentially investigated the “preventive effects” of ginsenosides on oxidative stress induced by rotenone, which was added to the cells after incubation with ginsenosides (line 99) “cells were pretreated with Rg1, Rg2, and Rd (20 μM) for 24 h prior to rotenone exposure (10 nM, 24 h)“.

Can you clarify, for example based on your own experiments, whether these substances would suppress oxidative stress and intracellular Ca²⁺ dysregulation in cells treated first with rotenone and then with ginsenosides? Would be the mechanism of pharmacological activity the same?

**Response:**

We thank you for this valuable comment regarding the therapeutic effects of ginsenosides. As clarified in the revised Methods section, ginsenosides were administered immediately before rotenone and remained present throughout the 24-h rotenone exposure. Therefore, the present study evaluated the protective pharmacological effects of ginsenosides during rotenone-induced cellular stress rather than post-treatment after oxidative injury had already been established. **Future studies comparing pre-, co-, and post-treatment protocols will be valuable for determining whether the underlying pharmacological responses differ under therapeutic conditions.**

6/ Authors stated limitations of their study, line 301: „SOD activity and IL-6 levels were measured to confirm rotenone-induced stress responses, the effects of ginsenosides on these markers were not systematically evaluated. „
This missing information would point to a link between oxidative stress parameters such as SOD activity, IL-6 concentrations and rotenone-associated Ca²⁺ dysregulation. Have similar analyses been described in other works?

**Response:**

Rotenone treatment was found to induce a simultaneous increase in intracellular Ca^2+^ and a decrease in SOD levels in neuronal cells (Hsu et al., 2021). In addition, the newly added MDA data in the present study further support the occurrence of rotenone-induced oxidative stress, complementing the SOD measurements. Although no previous study has directly demonstrated concurrent increases in intracellular Ca^2+^ and IL-6 following rotenone exposure, it is well established that IL-6 elevation in neurons enhances Ca^2+^ influx through NMDARs (Qiu et al., 1998), and that rotenone exacerbates dopaminergic neurotoxicity by increasing NMDAR-mediated Ca^2+^ influx (Wu and Johnson, 2011). Considering that rotenone upregulates IL-6 (Salari et al., 2023), these findings collectively suggest that rotenone-induced increases in intracellular Ca^2+^ and IL-6 may occur in parallel and potentially influence each other. Nevertheless, the present study specifically focused on elucidating the effects of ginsenosides on intracellular Ca^2+^ homeostasis. Therefore, we added this in Discussion section as follows.

Page 20:

However, previous studies have reported that rotenone increases intracellular Ca^2+^ levels while simultaneously decreasing SOD activity. In addition, rotenone activates NMDARs to increase Ca^2+^ influx, and elevated IL-6 levels have also been shown to activate NMDARs. Based on these findings, these findings collectively suggest that rotenone may promote Ca^2+^ influx through both a decrease in SOD activity and an increase in IL-6 levels.

7/ From the perspective of the application of ginsenosides in Parkinson's disease with existing pathology in the nervous system, do you assume the same mechanism of action as was observed in your in vitro study with selected experimental model?

**Response:**

We agree that the mechanisms operating in patients with Parkinson's disease may differ from those observed in our simplified in vitro model. The present study only confirmed the pharmacological response by measuring intracellular Ca^2+^ in neuronal and microglial cells in vitro. Therefore, our results merely represent pharmacological evidence. We agree that further in vivo, pharmacokinetic, and blood-brain barrier studies are needed. This point has been added to the discussion section.

Page 18:

Third, this study has a limitation that it is based on cell-based experimental results, which do not fully replicate the physiological environment of the human body.

We sincerely appreciate your thoughtful review, which has substantially improved the clarity, methodological transparency, and scientific interpretation of our manuscript. We hope that the revisions satisfactorily address all of the reviewer's concerns, and we sincerely appreciate the valuable comments, which have substantially improved the quality of our manuscript.

**References**

Hsu, S.-S., Lin, Y.-S., and Liang, W.-Z. (2021). Inhibition of the pesticide rotenone-induced Ca2+ signaling, cytotoxicity and oxidative stress in HCN-2 neuronal cells by the phenolic compound hydroxytyrosol. *Pesticide biochemistry and physiology* 179, 104979.

Ke, H., Bai, F., Li, Z., Zhu, Y., Zhang, C., Li, Y., et al. (2024). Inhibition of phospholipase D promotes neurological function recovery and reduces neuroinflammation after spinal cord injury in mice. *Frontiers in cellular neuroscience* 18, 1352630. doi: 10.3389/fncel.2024.1352630

Lee, D. K., In, J., and Lee, S. (2015). Standard deviation and standard error of the mean. *Korean journal of anesthesiology* 68, 220-223.

Mendez-Gomez, H. R., Singh, J., Meyers, C., Chen, W., Gorbatyuk, O. S., and Muzyczka, N. (2018). The lipase activity of phospholipase D2 is responsible for nigral neurodegeneration in a rat model of Parkinson’s disease. *Neuroscience* 377, 174-183. doi: 10.1016/j.neuroscience.2018.02.047.

Murphy, J. G., Sanderson, J. L., Gorski, J. A., Scott, J. D., Catterall, W. A., Sather, W. A., et al. (2014). AKAP-anchored PKA maintains neuronal L-type calcium channel activity and NFAT transcriptional signaling. *Cell reports* 7, 1577-1588. doi: 10.1016/j.celrep.2014.04.027

Ong, W.-Y., Farooqui, T., Koh, H.-L., Farooqui, A. A., and Ling, E.-A. (2015). Protective effects of ginseng on neurological disorders. *Frontiers in aging neuroscience* 7, 141694.

Qiu, Z., Sweeney, D. D., Netzeband, J. G., and Gruol, D. L. (1998). Chronic interleukin-6 alters NMDA receptor-mediated membrane responses and enhances neurotoxicity in developing CNS neurons. *Journal of Neuroscience* 18, 10445-10456.

Salari, Z., Ashabi, G., Fartoosi, A., Fartoosi, A., Shariatpanahi, M., Aghsami, M., et al. (2023). Sericin alleviates motor dysfunction by modulating inflammation and TrkB/BDNF signaling pathway in the rotenone-induced Parkinson’s disease model. *BMC Pharmacology and Toxicology* 24, 60.

Schmidt, S. I., Bogetofte, H., Ritter, L., Agergaard, J. B., Hammerich, D., Kabiljagic, A. A., et al. (2021). Microglia-secreted factors enhance dopaminergic differentiation of tissue-and iPSC-derived human neural stem cells. *Stem Cell Reports* 16, 281-294.

Sherer, T. B., Betarbet, R., Testa, C. M., Seo, B. B., Richardson, J. R., Kim, J. H., et al. (2003). Mechanism of toxicity in rotenone models of Parkinson's disease. *Journal of Neuroscience* 23, 10756-10764.

Siddiqui, R. A., and English, D. (1997). Phosphatidic acid elicits calcium mobilization and actin polymerization through a tyrosine kinase-dependent process in human neutrophils: a mechanism for induction of chemotaxis. *Biochimica et Biophysica Acta (BBA)-Lipids and Lipid Metabolism* 1349, 81-95.

Won, H.-J., Kim, H. I., Park, T., Kim, H., Jo, K., Jeon, H., et al. (2019). Non-clinical pharmacokinetic behavior of ginsenosides. *Journal of ginseng research* 43, 354-360.

Wu, Y.-N., and Johnson, S. (2011). Dopamine oxidation facilitates rotenone-dependent potentiation of N-methyl-D-aspartate currents in rat substantia nigra dopamine neurons. *Neuroscience* 195, 138-144.
