# Supplementary material for: Differential effects of ginsenosides on Ca2+ regulation in rotenone-treated neuronal and microglial cells
Source: PLoS One. 2026 Aug 18;21(8):e0356602. doi: 10.1371/journal.pone.0356602 (PMC13484979; doi:10.1371/journal.pone.0356602)
Supplement: Peer review history

# Peer review: Decision Letter 0

**DOI:** 10.1371/journal.pone.0356602.r001

**Authors:** Vinh Le Ba

25 Jun 2026

Differential effects of ginsenosides on Ca²⁺ regulation in rotenone-treated neuronal and microglial cells

PLOS One

Dear Dr. Seol,

Thank you for submitting your manuscript to PLOS ONE. After careful consideration, we feel that it has merit but does not fully meet PLOS ONE’s publication criteria as it currently stands. Therefore, we invite you to submit a revised version of the manuscript that addresses the points raised during the review process.

Please submit your revised manuscript by Jul 25 2026 11:59PM. If you will need more time than this to complete your revisions, please reply to this message or contact the journal office at plosone@plos.org. When you’re ready to submit your revision, log on to https://www.editorialmanager.com/pone/ and select the 'Submissions Needing Revision' folder to locate your manuscript file.

Please include the following items when submitting your revised manuscript:

- A letter that responds to each point raised by the academic editor and reviewer(s). You should upload this letter as a separate file labeled 'Response to Reviewers'.
- A marked-up copy of your manuscript that highlights changes made to the original version. You should upload this as a separate file labeled 'Revised Manuscript with Track Changes'.
- An unmarked version of your revised paper without tracked changes. You should upload this as a separate file labeled 'Manuscript'.

If you would like to make changes to your financial disclosure, please include your updated statement in your cover letter. Guidelines for resubmitting your figure files are available below the reviewer comments at the end of this letter.

If applicable, we recommend that you deposit your laboratory protocols in protocols.io to enhance the reproducibility of your results. Protocols.io assigns your protocol its own identifier (DOI) so that it can be cited independently in the future. For instructions see: https://journals.plos.org/plosone/s/submission-guidelines#loc-laboratory-protocols. Additionally, PLOS ONE offers an option for publishing peer-reviewed Lab Protocol articles, which describe protocols hosted on protocols.io. Read more information on sharing protocols at https://plos.org/protocols?utm_medium=editorial-email&utm_source=authorletters&utm_campaign=protocols.

As the corresponding author, your ORCID iD is verified in the submission system and will appear in the published article. PLOS supports the use of ORCID, and we encourage all coauthors to register for an ORCID iD and use it as well. Please encourage your coauthors to verify their ORCID iD within the submission system before final acceptance, as unverified ORCID iDs will not appear in the published article. *Only*  the individual author can complete the verification step; PLOS staff *cannot*  verify ORCID iDs on behalf of authors.

We look forward to receiving your revised manuscript.

Kind regards,

Vinh Le Ba, PhD

Academic Editor

PLOS ONE

Journal Requirements:

When submitting your revision, we need you to address these additional requirements.

1.Please ensure that your manuscript meets PLOS ONE's style requirements, including those for file naming. The PLOS ONE style templates can be found at

https://journals.plos.org/plosone/s/file?id=wjVg/PLOSOne_formatting_sample_main_body.pdf and

https://journals.plos.org/plosone/s/file?id=ba62/PLOSOne_formatting_sample_title_authors_affiliations.pdf

2. Thank you for stating the following financial disclosure:

“This work was supported by the National Research Foundation (NRF) of Korea grant funded by the Korea government (MSIT) (RS-2024-00353184).”

Please state what role the funders took in the study.  If the funders had no role, please state: 'The funders had no role in study design, data collection and analysis, decision to publish, or preparation of the manuscript.'

If this statement is not correct you must amend it as needed.

Please include this amended Role of Funder statement in your cover letter; we will change the online submission form on your behalf.

3. Thank you for stating the following in the Acknowledgments Section of your manuscript:

“This work was supported by the National Research Foundation (NRF) of Korea grant funded by the Korea government (MSIT) (RS-2024-00353184) and the Institute of Nursing Research, Korea University Grant. This work was conducted as part of the master’s thesis of JS at Korea University.”

We note that you have provided funding information that is not currently declared in your Funding Statement. However, funding information should not appear in the Acknowledgments section or other areas of your manuscript. We will only publish funding information present in the Funding Statement section of the online submission form.

Please remove any funding-related text from the manuscript and let us know how you would like to update your Funding Statement. Currently, your Funding Statement reads as follows:

“This work was supported by the National Research Foundation (NRF) of Korea grant funded by the Korea government (MSIT) (RS-2024-00353184).”

Please include your amended statements within your cover letter; we will change the online submission form on your behalf.

4. In the online submission form, you indicated that your data is available only on request from a third party. Please note that your Data Availability Statement is currently missing the name of the third party contact or institution / contact details for the third party, such as an email address or a link to where data requests can be made. Please update your statement with the missing information.

5. We note that you have included the phrase “data not shown” in your manuscript. Unfortunately, this does not meet our data sharing requirements. PLOS does not permit references to inaccessible data. We require that authors provide all relevant data within the paper, Supporting Information files, or in an acceptable, public repository. Please add a citation to support this phrase or upload the data that corresponds with these findings to a stable repository (such as Figshare or Dryad) and provide and URLs, DOIs, or accession numbers that may be used to access these data. Or, if the data are not a core part of the research being presented in your study, we ask that you remove the phrase that refers to these data.

6. If the reviewer comments include a recommendation to cite specific previously published works, please review and evaluate these publications to determine whether they are relevant and should be cited. There is no requirement to cite these works unless the editor has indicated otherwise.

Additional Editor Comments:

The reviewers identified a number of concerns that require substantial revision. In particular, the revised manuscript should:

• Provide more detailed methodological information to ensure reproducibility, including experimental design, cell culture conditions, cell seeding densities, treatment procedures, assay conditions, normalization approaches, and replication details.

• Clarify the experimental workflow, particularly the pretreatment design and the interpretation of the calcium measurements and inflammatory marker analyses.

• Strengthen the discussion of the proposed mechanisms involving calcium regulation, mitochondrial dysfunction, oxidative stress, and the potential roles of PLD- and PKA-related pathways, while avoiding conclusions that extend beyond the data presented.

• Address the reviewers’ concerns regarding statistical reporting, validation of key assays, biological significance of the observed effects, and the translational relevance of the findings.

Please provide a detailed point-by-point response to all reviewer comments and clearly indicate all changes made in the revised manuscript.

In view of the extent of the revisions required, I am inviting you to submit a revised version of your manuscript within 45 days.

I look forward to receiving your revised submission.

[Note: HTML markup is below. Please do not edit.]

Reviewer's Responses to Questions

**Comments to the Author**

1. Is the manuscript technically sound, and do the data support the conclusions?

Reviewer #1: Yes

Reviewer #2: Partly

Reviewer #3: Yes

Reviewer #4: Yes

**********

2. Has the statistical analysis been performed appropriately and rigorously?

Reviewer #1: Yes

Reviewer #2: I Don't Know

Reviewer #3: Yes

Reviewer #4: No

**********

3. Have the authors made all data underlying the findings in their manuscript fully available?

The PLOS Data policy

Reviewer #1: Yes

Reviewer #2: Yes

Reviewer #3: Yes

Reviewer #4: Yes

**********

4. Is the manuscript presented in an intelligible fashion and written in standard English?

Reviewer #1: No

Reviewer #2: Yes

Reviewer #3: Yes

Reviewer #4: Yes

**********

Reviewer #1: In this study, the authors tried to demonstrate the neuroprotective effects of ginsenosides against rotenone-induced mitochondrial toxicity. They proposed that PPT-type ginsenosides Rg1 and Rg2 mainly activate PLD-related signaling in both neuronal and microglial cells and also exhibit responses sensitive to LTCC in neurons. On the other hand, the PPD-type ginsenoside Rd demonstrated a broader range of pharmacological sensitivity, engaging PLD, LTCC, and PKA pathways. The persistent Ca²⁺ sensitivity to PLD inhibition in both cell types suggests that PLD-related pathways are involved in rotenone-induced Ca²⁺ dysregulation. This reviewer’s points are as follows:

1. This study requires minor paraphrasing and proofreading.

2. The present study seems like a short communication; the proper mechanistic overview is missing. The effect of ginsenoside on calcium homeostasis has been well demonstrated in mitochondrial toxicity assays, both in vitro and in vivo. However, the roles of PLD and PKA are novel. Therefore, they should highlight their novelty more strongly. Additionally, include more mechanistic data to establish a strong link between PLD and PKA-associated protective mechanisms.

Reviewer #2: The manuscript entitled "Differential effects of ginsenosides on Ca²⁺ regulation in rotenone-treated neuronal and microglial cells" presents interesting findings regarding the effects of ginsenosides on calcium homeostasis under rotenone-induced stress. However, substantial revisions are required to improve methodological transparency and to strengthen the validation of both the study concept and the reported results.

My primary concern is the lack of sufficient methodological detail, which currently limits reproducibility and makes it difficult for readers to critically evaluate the findings. Several essential experimental parameters are missing or insufficiently described, including:

The number of cells seeded per well for each experimental assay.

Whether experiments were performed in complete growth medium or under serum-free conditions. This is particularly important given the 24-hour pretreatment period followed by an additional 24-hour treatment period as understood from the MS.

Whether the culture medium was replaced after the pretreatment phase or whether ginsenosides remained present during the subsequent rotenone exposure. Clarification is needed to determine whether the effects reflect pretreatment alone or co-treatment with rotenone.

The calcium measurement protocol requires considerably more detail. Specifically, the authors should explain how intracellular Ca²⁺ levels were measured following 24 hours of treatment and a total of 48 hours of compound exposure. Since prolonged exposure may affect cell viability and cell number, it is unclear how the measured fluorescence signals can be attributed to specific changes in calcium regulation rather than differences in viable cell density. Information regarding assay normalization and controls is necessary.

In the Results section, Figure 1 raises several questions. If the red bars represent the same treatment concentration across all panels, the authors should explain why, in panels B and F, cell confluency decreases to approximately 60%, whereas cell viability remains between 80–90%. The apparent discrepancy between these measurements requires clarification.

The IL-6 measurements also require further validation and discussion. The authors should provide:

The detection limit, dynamic range, and sensitivity of the assay kit used. Information on positive and negative controls included in the assay. Justification for the biological significance of the observed IL-6 changes, particularly given that the reported concentrations appear very low (approximately 5 pg/mL) and the maximal induction is only about two-fold.

Overall, the study addresses an interesting topic; however, the current lack of methodological detail and insufficient validation of several key endpoints make it difficult to assess the robustness of the findings. Additional experimental details, clarification of the study design, and stronger justification of the biological significance of the reported effects are necessary before the conclusions can be fully evaluated.

Reviewer #3: This is an interesting paper that studies the effect of ginsenosides on Ca²⁺ regulation in rotenone-treated neurons/microglia cells. However, I have some comments for the authors.

1. Since you are trying to mimic Parkinson`s disease did you ensure that complex I was inhibited as you only treated the cells with rotenone and didn`t assay complex I activity?

2. I think more than one cell viability assay ids required in view of the limitations of the MTT assay. Did the authors assess any interference of the ginsenosides on the MTT assay. The full name of MTT is required before it is abbreviated as MTT.

3. I couldn`t find any evidence increased reactive oxygen species or oxidative stress measured in the paper or SOD levels and inflammatory markers. The authors should measure markers of oxidative stress such as Malondialdehyde or levels of cellular/mitochondrial oxidative stress.

4. Do the ginsenosides pass through the blood brain barrier as if not this would be a limitation in there use as a therapeutic agent?

5. Surely the loss of ATP in the rotenone treated cells would effect calcium homoeostasis and therefore in addition to the use of ginsenosides other agents that improve mitochondrial function such as coenzyme Q10 should be considered as dual treatments?

6. On a minor point, the abstract requires an introductory sentences to provide the relevance of the study.

Reviewer #4: Manuscript entitled “Differential effects of ginsenosides on Ca²⁺ regulation in rotenone-treated neuronal and microglial cells“ evaluated the effects of three structurally different ginsenosides on oxidative stress conditions induced by inhibitor of mitochondrial complex I.

Study showed new information about pharmacological activity of ginsenoside subtypes in rotenone-associated Ca²⁺ dysregulation in human neuronal SH-SY5Y cells and murine microglial BV2cell lines.

Comments:

1/ Justify why cell lines of different biological origin, i.e. human and mouse, were chosen. Could the observed differences in the effect of ginsenosides Rg1, Rg2, and Rd be related to the different biological origin of the cell lines?

2/ The abbreviations for ginsenosides appeared for the first time in the Abstract, but the full names of the individual compounds were missing. This needs to be added.

3/ Individual methodological procedures are described very briefly and lack information necessary for the possibility of reproducing in vitro experiments, for example, the number of cells used in individual tests and subsequent isolation from plastic.

4/ The authors used different numbers of replicates for each test and expressed results as mean ± SEM. Explain why the mean and SD, which are more statistically sensitive indicators of variability, were not calculated. Can you change calculations?

5/ The study essentially investigated the “preventive effects” of ginsenosides on oxidative stress induced by rotenone, which was added to the cells after incubation with ginsenosides (line 99) “cells were pretreated with Rg1, Rg2, and Rd (20 μM) for 24 h prior to rotenone exposure (10 nM, 24 h)“.

Can you clarify, for example based on your own experiments, whether these substances would suppress oxidative stress and intracellular Ca²⁺ dysregulation in cells treated first with rotenone and then with ginsenosides? Would be the mechanism of pharmacological activity the same?

6/ Authors stated limitations of their study, line 301: “SOD activity and IL-6 levels were measured to confirm rotenone-induced stress responses, the effects of ginsenosides on these markers were not systematically evaluated. “

This missing information would point to a link between oxidative stress parameters such as SOD activity, IL-6 concentrations and rotenone-associated Ca²⁺ dysregulation. Have similar analyses been described in other works?

7/ From the perspective of the application of ginsenosides in Parkinson's disease with existing pathology in the nervous system, do you assume the same mechanism of action as was observed in your in vitro study with selected experimental model?

**********

what does this mean?). If published, this will include your full peer review and any attached files.

If you choose “no”, your identity will remain anonymous but your review may still be made public.

**Do you want your identity to be public for this peer review?**  For information about this choice, including consent withdrawal, please see our Privacy Policy

Reviewer #1: No

Reviewer #2: No

Reviewer #3: No

Reviewer #4: No

**********

[NOTE: If reviewer comments were submitted as an attachment file, they will be attached to this email and accessible via the submission site. Please log into your account, locate the manuscript record, and check for the action link "View Attachments". If this link does not appear, there are no attachment files.]

To ensure your figures meet our technical requirements, please review our figure guidelines: https://journals.plos.org/plosone/s/figures

You may also use PLOS’s free figure tool, NAAS, to help you prepare publication quality figures: https://journals.plos.org/plosone/s/figures#loc-tools-for-figure-preparation.

NAAS will assess whether your figures meet our technical requirements by comparing each figure against our figure specifications.

# Author comment: Author response to Decision Letter 1

**DOI:** 10.1371/journal.pone.0356602.r002

27 Jul 2026

Response to the Editor

We sincerely thank the Editor for the careful evaluation of our manuscript and for providing constructive comments. We greatly appreciate the opportunity to revise our manuscript. Following the Editor's recommendations and the detailed comments from the four reviewers, we have substantially revised the manuscript to improve its clarity, methodological transparency, and scientific rigor. In response to the reviewers' major concerns, we not only extensively revised the manuscript but also performed additional experiments measuring malondialdehyde (MDA), a widely accepted marker of lipid peroxidation, to provide additional biochemical evidence of oxidative stress under rotenone treatment. These new experimental data further strengthen the biological basis of our conclusions and have been incorporated into the revised manuscript as a new main figure. All revisions are highlighted in the revised manuscript, and detailed point-by-point responses to each reviewer comment are provided below.

Journal Requirement #2

Comment

Please state what role the funders took in the study. If the funders had no role, please state: ‘The funders had no role in study design, data collection and analysis, decision to publish, or preparation of the manuscript.’

Response:

Thank you for this instruction. We have revised the Role of the Funder statement as requested. The revised Funding Statement now reads:

"This work was supported by the National Research Foundation (NRF) of Korea grant funded by the Korea government (MSIT) (RS-2024-00353184) and the Institute of Nursing Research, Korea University Grant. The funders had no role in study design, data collection and analysis, decision to publish, or preparation of the manuscript."

This statement has also been included in the Cover Letter so that the Funding Statement in the online submission system can be updated accordingly.

Journal Requirement #3

Comment

Funding information should not appear in the Acknowledgments section. Please update the Funding Statement accordingly.

Response:

Funding-related text has been removed from the Acknowledgments section in accordance with the Journal Requirements, and the revised Funding Statement has been provided in the Cover Letter for updating the online submission system.

Additional Editor Comments:

After careful evaluation, including comments from four independent reviewers, I believe that the manuscript addresses an interesting topic and presents potentially valuable findings regarding the effects of ginsenosides on calcium regulation under rotenone-induced cellular stress. However, several important issues must be addressed before the manuscript can be considered further for publication.

The reviewers identified a number of concerns that require substantial revision. In particular, the revised manuscript should:

• Provide more detailed methodological information to ensure reproducibility, including experimental design, cell culture conditions, cell seeding densities, treatment procedures, assay conditions, normalization approaches, and replication details.

Response:

We sincerely thank the Editor for the careful evaluation of our manuscript and for providing constructive comments. We greatly appreciate the opportunity to revise our manuscript. Following the Editor's recommendations and the detailed comments from the four reviewers, we have substantially revised the manuscript to improve its clarity, methodological transparency, and scientific rigor. In response to the reviewers' major concerns, we not only extensively revised the manuscript but also performed additional experiments measuring malondialdehyde (MDA), a widely accepted marker of lipid peroxidation, to provide additional biochemical evidence of oxidative stress under rotenone treatment. These new experimental data further strengthen the biological basis of our conclusions and have been incorporated into the revised manuscript as a new main figure together with the corresponding Results and Discussion.

All revisions are highlighted in the revised manuscript, and detailed point-by-point responses to each reviewer comment are provided below.

• Clarify the experimental workflow, particularly the pretreatment design and the interpretation of the calcium measurements and inflammatory marker analyses.

Response:

Thank you for this helpful comment. We have clarified the overall experimental workflow throughout the Methods and Results sections. The treatment protocol has been described more explicitly, including the timing of ginsenoside administration, the continuous presence of ginsenosides during rotenone exposure, and clarification that the experimental design reflects a co-treatment protocol rather than a distinct pretreatment phase. The calcium imaging protocol has also been clarified, including the distinction between ER Ca²⁺ release and extracellular Ca²⁺ influx, the rationale for AUC analysis, and normalization procedures. In addition, the rationale for measuring SOD activity and IL-6 as indicators of oxidative and inflammatory stress has been expanded, and the interpretation of these findings has been clarified in both the Results and Discussion sections. To improve transparency, the experimental timeline and treatment sequence have been revised to accurately describe the co-treatment protocol used throughout the study. We also clarified the rationale for selecting the rotenone treatment conditions used for the subsequent Ca²⁺ imaging experiments.

• Strengthen the discussion of the proposed mechanisms involving calcium regulation, mitochondrial dysfunction, oxidative stress, and the potential roles of PLD- and PKA-related pathways, while avoiding conclusions that extend beyond the data presented.

Response:

We agree with the Editor's recommendation. The Discussion has been extensively revised to better integrate our findings with previous literature regarding mitochondrial dysfunction, oxidative stress, intracellular Ca²⁺ regulation, and the potential involvement of PLD- and PKA-associated signaling pathways. To further strengthen the evidence for oxidative stress, we performed additional experiments measuring malondialdehyde (MDA), and these new data have been incorporated into the revised Results and Discussion sections. A new figure presenting the MDA results has been added to the revised manuscript to provide independent biochemical evidence supporting rotenone-induced oxidative stress and its attenuation by ginsenosides. We also revised several statements throughout the manuscript to avoid overinterpretation and to emphasize that our findings demonstrate pharmacological response profiles and pharmacological associations rather than direct molecular mechanisms. Furthermore, the Discussion now more clearly distinguishes experimentally supported findings from hypotheses that require future mechanistic investigation.

• Address the reviewers’ concerns regarding statistical reporting, validation of key assays, biological significance of the observed effects, and the translational relevance of the findings.

Response:

Thank you for this valuable suggestion. We have addressed all reviewer concerns regarding statistical analyses by providing additional methodological details, clarifying sample sizes and statistical procedures, and improving the presentation of the results. Additional information regarding assay validation, including assay sensitivity, dynamic range, and methodological limitations where appropriate. The Discussion has also been expanded to better explain the biological significance of the findings, their potential translational relevance, and the limitations of the present in vitro study, including issues related to blood-brain barrier permeability, pharmacokinetics, and future in vivo validation. Where appropriate, we also incorporated additional references suggested by the reviewers and revised the interpretation of our findings to better align with the scope of the experimental data. Collectively, these revisions improve both the methodological rigor and the biological interpretation of the study while maintaining conclusions that are fully supported by the experimental data.

Please provide a detailed point-by-point response to all reviewer comments and clearly indicate all changes made in the revised manuscript.

In view of the extent of the revisions required, I am inviting you to submit a revised version of your manuscript within 45 days.

I look forward to receiving your revised submission.

Once again, we sincerely thank the Editor and reviewers for their thoughtful comments, which have substantially improved the quality and clarity of our manuscript. We believe that the revised manuscript has been substantially strengthened through additional experimental validation, expanded methodological descriptions, improved transparency, and a more balanced interpretation of the findings. We have carefully addressed every comment raised by the Editor and reviewers in a point-by-point manner and have clearly indicated all revisions in the manuscript. We hope that the revised manuscript satisfactorily addresses all concerns and will now be suitable for further consideration for publication in PLOS ONE.

Review Comments to the Author

Please use the space provided to explain your answers to the questions above. You may also include additional comments for the author, including concerns about dual publication, research ethics, or publication ethics. (Please upload your review as an attachment if it exceeds 20,000 characters)

Reviewer #1:

In this study, the authors tried to demonstrate the neuroprotective effects of ginsenosides against rotenone-induced mitochondrial toxicity. They proposed that PPT-type ginsenosides Rg1 and Rg2 mainly activate PLD-related signaling in both neuronal and microglial cells and also exhibit responses sensitive to LTCC in neurons. On the other hand, the PPD-type ginsenoside Rd demonstrated a broader range of pharmacological sensitivity, engaging PLD, LTCC, and PKA pathways. The persistent Ca²⁺ sensitivity to PLD inhibition in both cell types suggests that PLD-related pathways are involved in rotenone-induced Ca²⁺ dysregulation. This reviewer’s points are as follows:

1. This study requires minor paraphrasing and proofreading.

Response:

Thank you for your insightful comments. We have carefully revised the manuscript through paraphrasing and proofreading to improve its overall quality. In addition, we revised the title, Abstract, Introduction, Discussion, and figure legends to improve clarity, consistency in terminology, and scientific precision throughout the manuscript. Several statements were also rephrased to avoid overinterpretation and to ensure that the conclusions are fully supported by the experimental data.

2. The present study seems like a short communication; the proper mechanistic overview is missing. The effect of ginsenoside on calcium homeostasis has been well demonstrated in mitochondrial toxicity assays, both in vitro and in vivo. However, the roles of PLD and PKA are novel. Therefore, they should highlight their novelty more strongly. Additionally, include more mechanistic data to establish a strong link between PLD and PKA-associated protective mechanisms.

Response:

Thank you for recognizing the novelty of our work. We specifically focused on phospholipase D (PLD) and protein kinase A (PKA) to evaluate the pharmacological sensitivity of ginsenosides. Previous studies have implicated PLD in neuronal degeneration and inflammatory responses in neurodegenerative diseases, and its product phosphatidic acid (PA) functions as a second messenger for Ca²⁺ mobilization (Siddiqui and English, 1997; Mendez-Gomez et al., 2018; Ke et al., 2024). PKA regulates L-type Ca²⁺ channel (LTCC)-mediated Ca²⁺ influx (Murphy et al., 2014). Based on these findings, we investigated whether ginsenosides could modulate rotenone-induced intracellular Ca²⁺ dysregulation through PLD- and PKA-associated pharmacological pathways.

We agree that direct mechanistic evidence would further strengthen our conclusions. However, the primary objective of the present study was to compare the pharmacological response profiles of structurally distinct ginsenosides using selective pharmacological inhibitors rather than to identify direct molecular targets. Accordingly, throughout the revised manuscript, we consistently describe these findings as pharmacological response profiles inferred from inhibitor-based analyses rather than direct molecular mechanisms.

In addition, to strengthen the biological relevance of the rotenone model, we performed new experiments measuring malondialdehyde (MDA), a marker of lipid peroxidation, and incorporated these data into the revised manuscript. The new MDA results complement the SOD findings and provide independent biochemical evidence supporting rotenone-induced oxidative stress.

Accordingly, we revised the Introduction and Discussion to better highlight the novelty of the pharmacological findings while clearly acknowledging the limitations of the current inhibitor-based approach.

Reviewer #2:

The manuscript entitled "Differential effects of ginsenosides on Ca²⁺ regulation in rotenone-treated neuronal and microglial cells" presents interesting findings regarding the effects of ginsenosides on calcium homeostasis under rotenone-induced stress. However, substantial revisions are required to improve methodological transparency and to strengthen the validation of both the study concept and the reported results.

1. My primary concern is the lack of sufficient methodological detail, which currently limits reproducibility and makes it difficult for readers to critically evaluate the findings. Several essential experimental parameters are missing or insufficiently described, including:

The number of cells seeded per well for each experimental assay.

Whether experiments were performed in complete growth medium or under serum-free conditions. This is particularly important given the 24-hour pretreatment period followed by an additional 24-hour treatment period as understood from the MS.

Whether the culture medium was replaced after the pretreatment phase or whether ginsenosides remained present during the subsequent rotenone exposure. Clarification is needed to determine whether the effects reflect pretreatment alone or co-treatment with rotenone.

Response:

We sincerely thank you for the thoughtful and constructive review. We agree that additional methodological detail is necessary to ensure reproducibility. Accordingly, we have substantially revised the methods section. We have also clarified the experimental protocol and calcium measurement procedure to improve methodological transparency and reproducibility.

All treatments in our experiments were conducted in complete growth medium containing 10% fetal bovine serum (FBS), and we added this information to the Methods section.

Regarding the treatment protocol, ginsenosides Rg1, Rg2, or Rd were added to the culture medium immediately before rotenone administration. Rotenone was then added directly into the same ginsenoside-containing medium without replacement, and cells were incubated with both compounds for 24 h. Therefore, the observed effects reflect a co-treatment protocol rather than a distinct pretreatment phase. We apologize for the earlier description implying a 24-h pretreatment followed by an additional 24-h treatment period, and we have revised the Methods section to accurately reflect the actual experimental protocol. All corresponding descriptions in the Methods, Results, figure legends, and Discussion have been revised to ensure consistency with the actual experimental protocol.

For comparative pharmacological analysis, cells were treated with ginsenosides Rg1, Rg2, and Rd (20 µM) immediately before rotenone (10 nM) administration. Rotenone was then added directly into the same ginsenoside-containing medium without replacement, and cells were co-incubated with both compounds for 24 h in complete growth medium.

We have also added the number of cells seeded for each experimental assay in the Methods section.

2. The calcium measurement protocol requires considerably more detail. Specifically, the authors should explain how intracellular Ca²⁺ l

Attachment Submitted filename: Response to the Editor and Reviewers_072426 TO7(2).docx

# Editor report: Decision Letter 1

**DOI:** 10.1371/journal.pone.0356602.r003

**Authors:** Vinh Le Ba

5 Aug 2026

Differential effects of ginsenosides on Ca²⁺ regulation in rotenone-treated neuronal and microglial cells

PONE-D-26-22746R1

Dear Dr. Seol,

We’re pleased to inform you that your manuscript has been judged scientifically suitable for publication and will be formally accepted for publication once it meets all outstanding technical requirements.

Within one week, you’ll receive an e-mail detailing the required amendments. When these have been addressed, you’ll receive a formal acceptance letter and your manuscript will be scheduled for publication.

An invoice will be generated when your article is formally accepted. Please note, if your institution has a publishing partnership with PLOS and your article meets the relevant criteria, all or part of your publication costs will be covered. Please make sure your user information is up-to-date by logging into Editorial Manager at Editorial Manager® and clicking the ‘Update My Information' link at the top of the page. For questions related to billing, please contact billing support.

If your institution or institutions have a press office, please notify them about your upcoming paper to help maximize its impact. If they’ll be preparing press materials, please inform our press team as soon as possible -- no later than 48 hours after receiving the formal acceptance. Your manuscript will remain under strict press embargo until 2 pm Eastern Time on the date of publication. For more information, please contact onepress@plos.org.

Kind regards,

Vinh Le Ba, PhD in Pharmaceutical Science

Academic Editor

PLOS One

Additional Editor Comments (optional):

The authors have carefully addressed the reviewers' comments and provided a well-revised manuscript. The revised version is suitable for publication in PLOS ONE. Therefore, I recommend accepting the manuscript in its current form.

Reviewers' comments:

# Editor report: Acceptance letter

**DOI:** 10.1371/journal.pone.0356602.r004

**Authors:** Vinh Le Ba

PONE-D-26-22746R1

PLOS One

Dear Dr. Seol,

I'm pleased to inform you that your manuscript has been deemed suitable for publication in PLOS One. Congratulations! Your manuscript is now being handed over to our production team.

At this stage, our production department will prepare your paper for publication. This includes ensuring the following:

* All references, tables, and figures are properly cited

* All relevant supporting information is included in the manuscript submission,

* There are no issues that prevent the paper from being properly typeset

You will receive further instructions from the production team, including instructions on how to review your proof when it is ready. Please keep in mind that we are working through a large volume of accepted articles, so please give us a few days to review your paper and let you know the next and final steps.

Lastly, if your institution or institutions have a press office, please let them know about your upcoming paper now to help maximize its impact. If they'll be preparing press materials, please inform our press team within the next 48 hours. Your manuscript will remain under strict press embargo until 2 pm Eastern Time on the date of publication. For more information, please contact onepress@plos.org.

You will receive an invoice from PLOS for your publication fee after your manuscript has reached the completed accept phase. If you receive an email requesting payment before acceptance or for any other service, this may be a phishing scheme. Learn how to identify phishing emails and protect your accounts at https://explore.plos.org/phishing.

If we can help with anything else, please email us at customercare@plos.org.

Thank you for submitting your work to PLOS ONE and supporting open access.

Kind regards,

PLOS ONE Editorial Office Staff

on behalf of

Dr. Vinh Le Ba

Academic Editor

PLOS One
